# Effect of testosterone therapy on breast tissue composition and mammographic breast density in trans masculine individuals

**DOI:** 10.1186/s13058-024-01867-w

**Published:** 2024-07-02

**Authors:** Yujing J. Heng, Gabrielle M. Baker, Valerie J. Fein-Zachary, Yaileen D. Guzman-Arocho, Vanessa C. Bret-Mounet, Erica S. Massicott, Vanda F. Torous, Stuart J. Schnitt, Sy Gitin, Paul Russo, Adam M. Tobias, Richard A. Bartlett, Gopal Varma, Despina Kontos, Lusine Yaghjyan, Michael S. Irwig, Jennifer E. Potter, Gerburg M. Wulf

**Affiliations:** 1 Department of Pathology, Beth Israel Deaconess Medical Center, Harvard Medical School, 330 Brookline Ave, Dana 517B, Boston, MA 02115 USA; 2 Department of Radiology, Beth Israel Deaconess Medical Center, Harvard Medical School, Boston, MA USA; 3 Department of Pathology, Massachusetts General Hospital, Harvard Medical School, Boston, MA USA; 4 Dana-Farber/Brigham and Women’s Cancer Center, Dana-Farber Cancer Institute-Brigham and Women’s Hospital, Harvard Medical School, Boston, MA USA; 5 The Fenway Institute, Boston, MA USA; 6 Department of Surgery, Beth Israel Deaconess Medical Center, Harvard Medical School, Boston, MA USA; 7 Departments of Radiology, Biomedical Informatics, and Biomedical Engineering, Columbia University Irving Medical Center, New York, NY USA; 8 Department of Epidemiology, College of Public Health and Health Professions and College of Medicine, University of Florida, Gainesville, FL USA; 9 Department of Medicine, Beth Israel Deaconess Medical Center, Harvard Medical School, Boston, MA USA

**Keywords:** Gender-affirming hormones, Transgender health disparities, Breast cancer risk, Trans men, Non binary people, Hormone replacement therapy, Top surgery

## Abstract

**Background:**

The effect of gender-affirming testosterone therapy (TT) on breast cancer risk is unclear. This study investigated the association between TT and breast tissue composition and breast tissue density in trans masculine individuals (TMIs).

**Methods:**

Of the 444 TMIs who underwent chest-contouring surgeries between 2013 and 2019, breast tissue composition was assessed in 425 TMIs by the pathologists (categories of lobular atrophy and stromal composition) and using our automated deep-learning algorithm (% epithelium, % fibrous stroma, and % fat). Forty-two out of 444 TMIs had mammography prior to surgery and their breast tissue density was read by a radiologist. Mammography digital files, available for 25/42 TMIs, were analyzed using the LIBRA software to obtain percent density, absolute dense area, and absolute non-dense area. Linear regression was used to describe the associations between duration of TT use and breast tissue composition or breast tissue density measures, while adjusting for potential confounders. Analyses stratified by body mass index were also conducted.

**Results:**

Longer duration of TT use was associated with increasing degrees of lobular atrophy (*p* < 0.001) but not fibrous content (*p* = 0.82). Every 6 months of TT was associated with decreasing amounts of epithelium (exp(β) = 0.97, 95% CI 0.95,0.98, adj *p* = 0.005) and fibrous stroma (exp(β) = 0.99, 95% CI 0.98,1.00, adj *p* = 0.05), but not fat (exp(β) = 1.01, 95%CI 0.98,1.05, adj *p* = 0.39). The effect of TT on breast epithelium was attenuated in overweight/obese TMIs (exp(β) = 0.98, 95% CI 0.95,1.01, adj *p* = 0.14). When comparing TT users versus non-users, TT users had 28% less epithelium (exp(β) = 0.72, 95% CI 0.58,0.90, adj *p* = 0.003). There was no association between TT and radiologist’s breast density assessment (*p* = 0.58) or LIBRA measurements (*p* > 0.05).

**Conclusions:**

TT decreases breast epithelium, but this effect is attenuated in overweight/obese TMIs. TT has the potential to affect the breast cancer risk of TMIs. Further studies are warranted to elucidate the effect of TT on breast density and breast cancer risk.

**Supplementary Information:**

The online version contains supplementary material available at 10.1186/s13058-024-01867-w.

## Introduction

About 65% of trans masculine individuals (TMIs) pursue testosterone therapy (TT) to treat their gender dysphoria [1]. TMIs are defined in this paper as individuals who were born female and identify as trans men or non-binary people. The breast is highly sensitive to sex hormones. Normal breast proliferation is regulated by the balance between the stimulating effects of estrogens and the inhibitory effects of testosterone. TT increases the baseline testosterone levels of TMIs by ≥ tenfold to achieve levels comparable to cisgender men [2]. The extent to which TT affects breast tissue and breast cancer (BC) risk remains unclear.

While TT has been explored to prevent and/or treat BC in cisgender women [3–6], epidemiological studies in cisgender women reported an association between high circulating testosterone levels and increased BC risk [7–12]. One explanation for that phenomenon is that testosterone can be aromatized to estradiol, contributing to breast cell proliferation and tumorigenesis [13]. In contrast, three epidemiological studies that examined BC incidence in trans people concluded that trans men do not have an increased BC risk compared to cisgender females [14–16].

Most trans masculine breast-related studies to date are histopathological reviews of breast tissues obtained after chest-contouring surgeries. We and others have shown that TT has a profound effect on non-cancer breast morphology [17–23]. Our group observed that alterations in breast histology, in particular, higher degrees of lobular atrophy, were evident after ≥ 12 months of TT [23]. We also subsequently reported that that Toker cell hyperplasia, a histological feature with unclear clinical significance, was more frequently observed in TMIs compared to cisgender women [24].

Mammographic breast density reflects the relative amounts of breast fibroglandular (epithelium and stroma) and fat. High mammographic breast density is a strong BC risk factor for cisgender women [25]. We previously developed a computational pathology algorithm to assess non-cancer breast tissue composition [26–29]. Using our algorithm, women with more breast epithelium (highest quartile) had higher subsequent BC risk compared with women in the lowest quartile [28]. Hormone-related BC risk factors such as reproductive and early body weight were also linked to changes in breast tissue composition [26, 27]. Thus, it is important to understand the effect of TT on breast tissue composition in order to understand trans masculine BC risk.

To address these knowledge gaps, we investigated the association of TT and breast tissue composition in 425 TMIs. In a subset of 42 subjects who had mammograms, we explored the relationship between TT and mammographic breast density, and performed radiology-pathology correlations.

## Materials and methods

### Study subjects

**Fig. 1:**
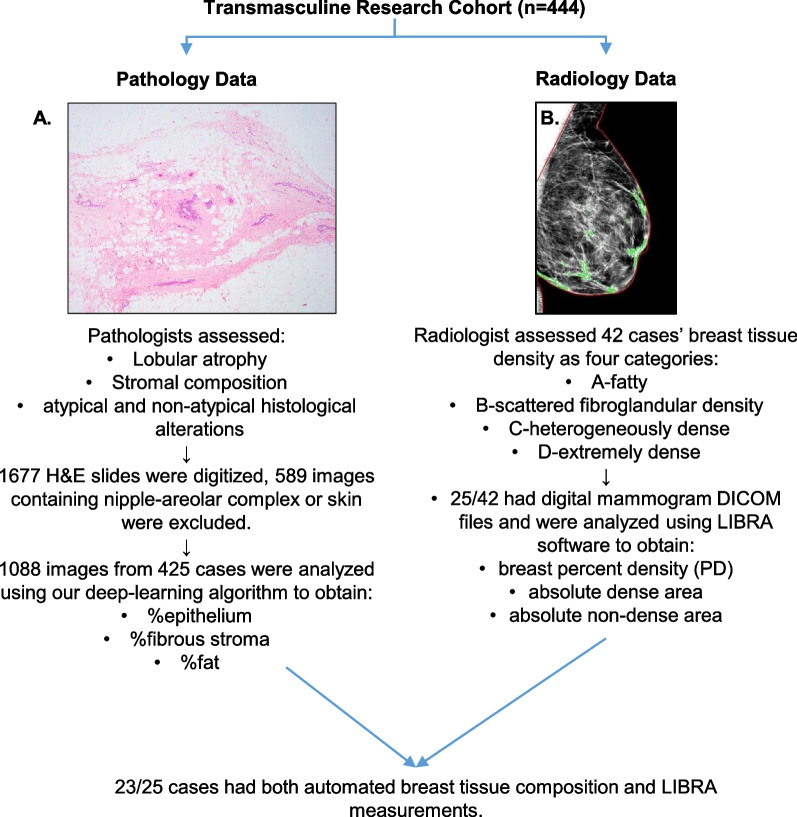
Summary of the histological, radiological, and quantitative data used for each part of this study. **a** Breast histology image from a subject who had been using testosterone therapy for 4.2 years at the time of chest-contouring surgery. Pathologists assessed the breast tissue as demonstrating a moderate degree of lobular atrophy with mixed fatty and fibrous stroma. Our algorithm quantified this breast tissue as containing 2.8% epithelium, 53.1% fibrous stroma, and 44.1% fat. **b** Corresponding mammogram from the same subject taken two months prior to surgery. The radiologist classified this case as B-scattered fibroglandular density. The Laboratory for Individualized Breast Radiodensity Assessment (LIBRA) software estimated the breast percent density as 1.9%. Digital imaging and communications in medicine, DICOM

This cross-sectional study initially included 444 TMIs who had chest-contouring surgery at an urban medical center between 2013 and 2019 [23, 30]. Clinical data were retrieved from medical record, including age at surgery, race/ethnicity, family history of BC, parity, oophorectomy status at time of surgery, body mass index (BMI), alcohol consumption, TT regimen, duration of TT (months), and whether they bound their chest. We estimated the duration of TT at time of surgery in one of two ways depending on data availability: (1) by calculating the number of months between date of the first TT prescription and date of surgery, or (2) by combining the subject’s verbal recount of how long they had been receiving TT at their pre-surgical consult and the time between that pre-surgical consult and date of surgery. We do not have circulating estradiol levels as clinicians do not routinely monitor estradiol levels in TMIs taking testosterone. One subject was taking letrozole, an aromatase inhibitor. No subject was taking ovarian suppression with luteinizing hormone-releasing hormone agonists. Figure 1 summarizes the histological, radiological, and quantitative data used in this study. The study was approved by the BIDMC Institutional Review Board (2018P000814).

### Pathological review and automated breast tissue composition

**Fig. 2:**
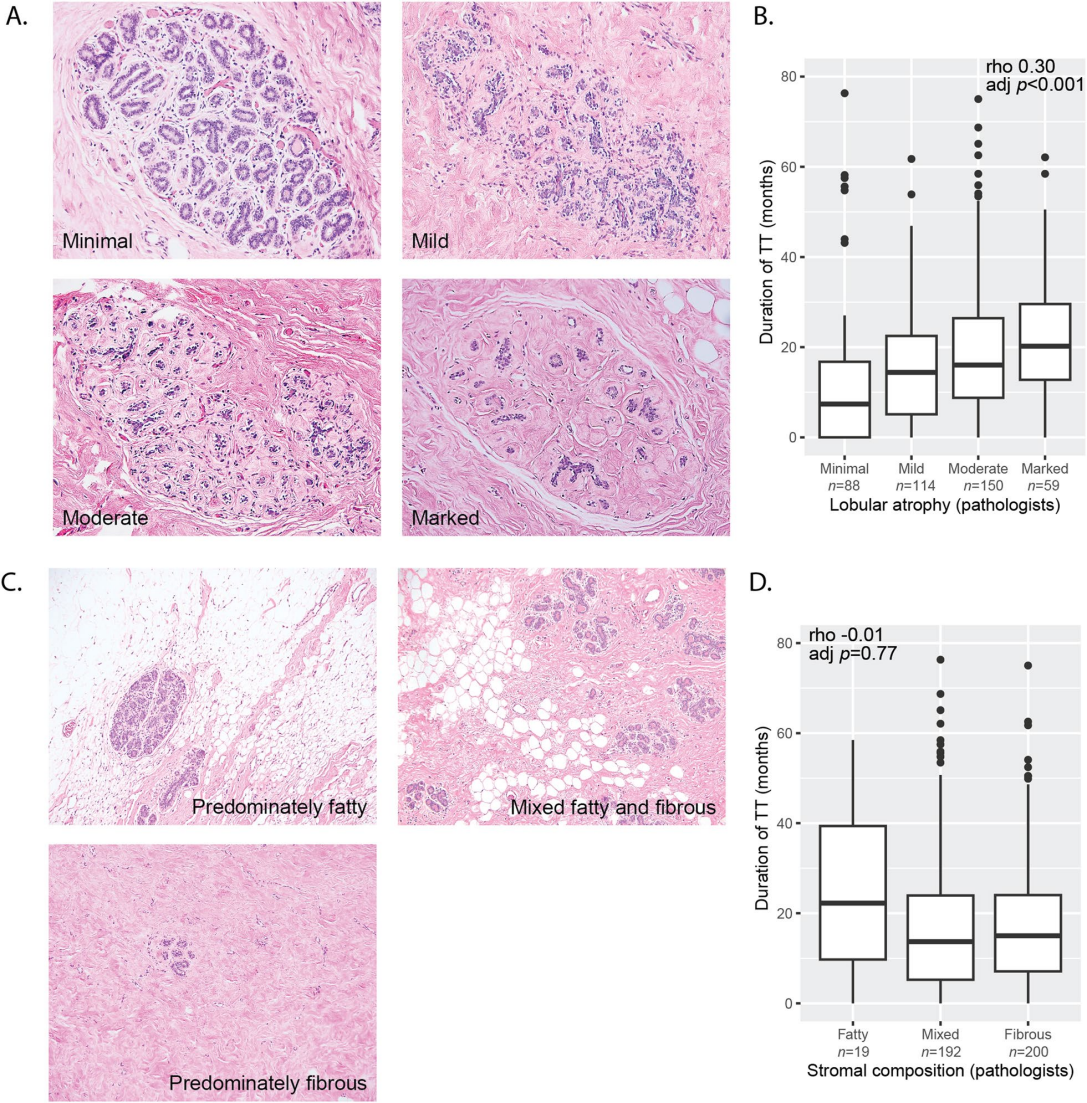
**a** Lobular atrophy was assessed by the pathologists using four categories. **b** The duration of testosterone therapy (TT) significantly correlated with increasing degrees of lobular atrophy (rho = 0.30, 95% Confidence Interval (CI) 0.21, 0.38, adj *p* < 0.001). **c** Stromal composition was assessed by the pathologists using three categories. **d** There was no correlation between duration of TT and stromal composition as assessed by the pathologists (rho = − 0.01, 95% CI − 0.11,0.08, adj *p* = 0.77)

**Fig. 3:**
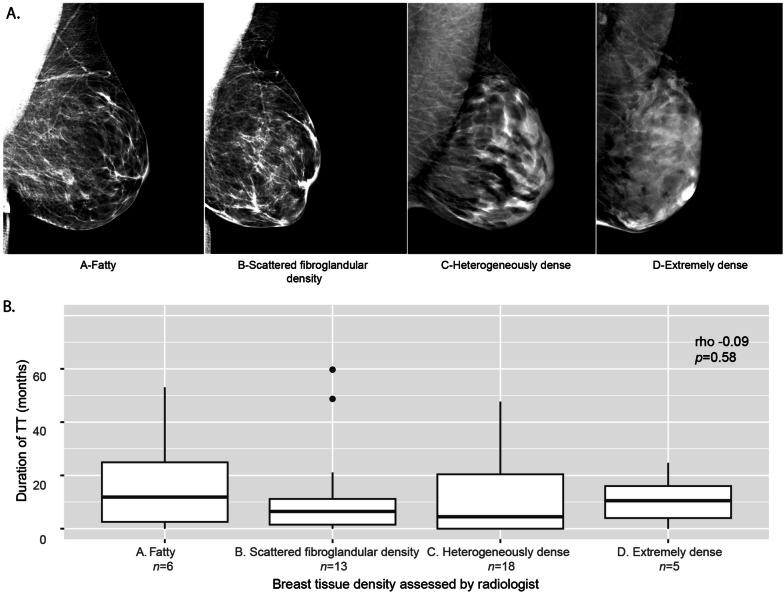
**a** Breast tissue density was assessed by a radiologist using four categories with increasing amounts of fibroglandular tissue (epithelium and fibrous stroma). Mammograms were from transmasculine subjects. **b** There was no association between the duration of testosterone therapy (TT) and breast tissue density (rho = − 0.09, 95% Confidence Interval (CI) − 0.38, 0.22, *p* = 0.58)

The pathology department’s grossing protocol for chest-contouring specimens was to sample each quadrant of the breast parenchyma, and submit two blocks per breast. Additional sections were submitted if the nipple or skin was present, and if a gross lesion or atypia was identified [23]. For each of the 444 cases, H&E-stained slides were reviewed by two pathologists (VT and SJS [22], or GMB and YDG [23]). Each case was assessed for (1) degree of lobular atrophy classified as minimal, mild, moderate, or marked (Fig. 2a), (2) stromal composition classified as predominantly fatty, mixed fatty and fibrous, and predominantly fibrous (Fig. 2c), and (3) atypical and non-atypical histological alterations [23]. Atypical breast lesions included ductal carcinoma in situ (DCIS), lobular carcinoma in situ, atypical ductal hyperplasia (ADH), atypical lobular hyperplasia (ALH), and flat epithelial atypia. Slides (*n* = 1677) were digitized at 20 × using the Pannoramic Scan P150 (3DHISTECH Ltd, Budapest, Hungary).

We previously developed a deep-learning algorithm to segment breast histological images into epithelium, fibrous stroma, and fat [28]. As this algorithm was not developed using images containing nipple-areolar complex or skin, it was likely to erroneously classify pixels containing nipple-areolar complex or skin as breast epithelium. Hence, we excluded images with nipple-areolar complex or skin (*n* = 589) and applied the algorithm to the remaining 1,088 images from 425 transmasculine subjects. The number of pixels classified as epithelium, fibrous stroma, or fat, were summed across the images of each subject, divided by the total number of tissue pixels detected across all images, and expressed as a percentage (%). Automated breast tissue composition hereafter refers to quantitative % epithelium, % fibrous stroma, and % fat obtained using histological images and our algorithm.

### Radiological assessment and quantitative measures of mammographic breast density

A subset of 42 out of 444 (9.5%) TMIs had mammograms prior to surgery. Additional clinical data were retrieved to reflect oophorectomy status, duration of TT, and chest binding at the time of mammogram. BMI at time of mammogram was not available. The radiologist (VJF) assessed breast tissue density by classifying the densest area as A-fatty, B-scattered fibroglandular density, C-heterogeneously dense, or D-extremely dense.

Digital mammography files in digital imaging and communications in medicine (DICOM) format were available for 25 out of 42 subjects (59.5%). DICOM files for the other 17 subjects were unavailable as their mammograms were conducted elsewhere. DICOM files were processed using the fully automated, publicly available Laboratory for Individualized Breast Radiodensity Assessment (LIBRA) software [31]. LIBRA measurements correlate to Cumulus, an established research software, and Volpara, a commercially available software [32]. LIBRA identifies the breast region on the mammogram, partitions the breast into gray-level intensity clusters which are then aggregated into the final dense tissue segmentation, and calculates the area of dense pixels to estimate the total absolute dense area. Breast percent density (PD) is obtained by normalizing the absolute dense area by the total breast area [31–33]. Absolute non-dense area is calculated by subtracting absolute dense area from total breast area. Since subjects can have up to two mediolateral oblique views per breast, each LIBRA measurement was averaged across multiples DICOM files of the right and left breast. LIBRA measurements hereafter refer to the quantitative values of PD, absolute dense area, and absolute non-dense area.

### Statistical analysis

Mann–Whitney or Fisher’s exact test was used to compare clinical characteristics. Spearman’s rho was used to describe the correlations between pathologists’ assessments and automated breast tissue composition measures, overall and stratified by TT; 95% confidence interval (CI) was calculated using the RVAideMemoire package in R. Partial correlation analysis was used to relate the duration of TT (months; continuous variable) to the pathologists’ assessments (ordinal variables), adjusting for age at surgery (psych package, R).

Linear regression modeled the relationship between every 6 months of TT and automated breast tissue composition (natural log-transformed), adjusting for: (1) age and year of surgery (model 1); or (2) age, year of surgery, race/ethnicity, BMI at surgery, chest binding, and oophorectomy (model 2). Non-TT users were assigned as zero months of TT in the linear regression analyses. To control for differences in TT dosages, we additionally controlled for estimated weekly testosterone (mg) to clarify the effect of dose on tissue composition (model 3). Optimal TT dosages are not solely determined by circulating plasma levels but are frequently adjusted for patients to achieve their desired masculinizing effect. TMIs may receive daily, weekly, or biweekly TT, depending on the mode of administration (transdermal gel/patch, subcutaneous pellet implant, subcutaneous or intra-muscular injection) [30]. We performed sensitivity analyses by: (1) additionally controlling for alcohol consumption; (2) excluding cases with atypical lesions; (3) restricting to TT-users that administered testosterone via intra-muscular injection and non-TT users; and (4) restricting to nulliparous subjects. We also performed analyses stratified by BMI (normal weight BMI < 25 and overweight/obese BMI ≥ 25). Secondary analysis evaluated the relationship between TT use (users versus non-users) and breast tissue composition. To improve the interpretability of the beta coefficient, we back transformed the natural log beta coefficients.

For 42 subjects who had mammography prior to their surgeries, we used Spearman’s rho to explore the correlation between TT and breast tissue density assessed by the radiologist, and stratified by age (< 40 and ≥ 40 years old). Among the 25 subjects with DICOM files, we correlated the radiologist’s assessment and LIBRA measurements using Spearman’s rho. The association of TT duration (per 6 months) with each LIBRA measurement (natural log-transformed) was assessed using linear regression (without adjustment and adjusted for age and BMI at surgery); we also stratified by BMI (< 25 and ≥ 25) and restricted to nulliparous subjects.

Twenty three out of 25 subjects had both histological images and DICOM files allowing for correlative analyses using Spearman’s rho. Sensitivity analysis was conducted by restricting to 17 out of 23 individuals who had surgeries within 6 months after their mammography. Analyses were conducted using R. The level of significance for all statistical tests (2-sided) was *p* < 0.05.

## Results

### Subjects with pathology data

The age range of 425 TMIs who had breast tissue composition data was 18–61 years old (median = 25), and 326 TMIs (76.7%) were White. Three hundred and fifty-seven subjects used TT (84.0%) and 38.9% used TT for one to two years (Table 1). Among the TT users, 312 out of 357 (87.4%) administered TT via intra-muscular injection, 5 (1.4%) via subcutaneous pellet, 28 (7.8%) via transdermal gel/patch, and for 12 (3.4%) mode of administration was unknown. TT users were also two years younger (*p* = 0.003), and more likely to bind their chest (*p* = 0.03) compared to non-users. Atypical breast lesions were only detected among TT users (*p* = 0.03): 1 case with DCIS, 5 cases with ADH, 2 cases with ALD, and 1 case had both ADH and ALH. Non-TT users were more likely to consume alcohol (*p* = 0.01). There was no difference in family history of BC, parity, oophorectomy status, or BMI, between TT users and non-users (*p* > 0.05; Table 1).

**Table 1:** Characteristics of the 425 transmasculine subjects, stratified by testosterone therapy (TT) use

	Used TT	Did not use TT	*p* value
	*n* = 357	*n* = 68	
Age at surgery, median [IQR]	25.0 [21.0, 29.0]	27.0 [23.8, 32.2]	0.003^a^
Race/ethnicity, *n* (%)			0.47^b^
White	277 (77.6)	49 (72.1)	
Black or African American	30 (8.4)	6 (8.8)	
Asian	20 (5.6)	1 (1.5)	
Multiracial	18 (5.0)	4 (5.9)	
Native American/Pacific Islander	2 (0.6)	1 (1.5)	
Unspecified	10 (2.8)	7 (10.3)	
Family history of breast cancer, *n* (%)			0.63^b^
Yes	94 (26.3)	18 (26.5)	
No	207 (58.0)	34 (50.0)	
Not reported	56 (15.7)	16 (23.5)	
Parity, *n* (%)			0.41^b^
Parous	8 (2.2)	3 (4.4)	
Nulliparous	142 (39.8)	29 (42.6)	
Not reported	207 (58.0)	36 (52.9)	
Oophorectomy prior to surgery, *n* (%)			0.22^b^
Yes	20 (5.6)	1 (1.5)	
No	337 (94.4)	67 (98.5)	
Breast lesions, *n* (%)			0.03^b^
Atypical lesions	9 (2.5)	0 (0.0)	
Benign lesions	290 (81.2)	64 (94.1)	
None	58 (16.2)	4 (5.9)	
BMI at surgery, median [IQR]	25.8 [23.2, 29.9]	25.4 [23.0, 29.5]	0.57^a^
Alcohol consumption, *n* (%)			0.01^b^
Current	186 (52.1)	48 (70.6)	
Never	159 (44.5)	20 (29.4)	
Not reported	12 (3.4)	0 (0.0)	
Duration of TT at surgery, *n* (%)			–
Never	0 (0.0)	68 (100.0)	
< 1 year	93 (26.1)	0 (0.0)	
> = 1 to < 2 years	139 (38.9)	0 (0.0)	
> = 2 to < 5 years	97 (27.2)	0 (0.0)	
> = 5 years	14 (3.9)	0 (0.0)	
Unspecified duration	14 (3.9)	0 (0.0)	
Chest binding, *n* (%)			0.03^b^
Yes	169 (47.3)	20 (29.4)	
No	15 (4.2)	6 (8.8)	
Not reported	173 (48.5)	42 (61.8)	

*p*-values were obtained using the ^a^Mann-Whitney or ^b^Fisher’s exact test. Data in “not reported or unspecified” categories were excluded from statistical analysis. Body mass index, BMI; Inter-quartile range, IQR. Percentages may not add up to 100% due to rounding

### Association of TT and breast tissue composition

The duration of TT use was significantly correlated with increasing degrees of lobular atrophy (rho = 0.30, 95% CI 0.21,0.38, adj *p* < 0.001; Fig. 2b) but was not correlated with fibrous content of the stroma (rho = − 0.01, 95% CI − 0.11,0.08, adj *p* = 0.77; Fig. 2d) as assessed by the pathologists. Algorithm-derived breast tissue composition data significantly correlated with pathologists’ assessments (all *p* < 0.001; Additional file 1: 1).

For every 6 months of TT use, the amount of breast epithelium decreased by 3% in the fully adjusted model 3 (exp(β) = 0.97, 95% CI 0.95,0.98, adj *p* = 0.005; Table 2). The amount of fibrous stroma also decreased by 1% per 6 months of TT (model 3, exp(β) = 0.99, 95% CI 0.98,1.00, adj *p* = 0.05; Table 2). Although % fat increased by 2% for every 6 months of TT use (model 1 exp(β) = 1.02, 95% CI 1.00,1.04, adj *p* = 0.01; Table 2), this association did not hold up when adjusted for variables in models 2 and 3.

**Table 2:** The association of testosterone therapy (per six months duration) and the percentages (%) of each breast tissue region

	*n*	Exp(β)	95% CI	*p* value
% epithelium				
Model 1	411	0.97	0.95–0.98	< 0.001
Model 2	207	0.96	0.94–0.98	0.001
Model 3	193	0.97	0.95–0.99	0.005
% fibrous stroma				
Model 1	411	0.99	0.98–0.99	< 0.001
Model 2	207	0.99	0.98–1.00	0.02
Model 3	193	0.99	0.98–1.00	0.05
% fat				
Model 1	411	1.02	1.00–1.04	0.01
Model 2	207	1.02	0.99–1.06	0.15
Model 3	193	1.01	0.98–1.05	0.39

Model 1 adjusted for age and year of surgery. Model 2 adjusted for age and year of surgery, race/ethnicity, BMI, chest binding, and oophorectomy status. Model 3 adjusted for age and year of surgery, race/ethnicity, BMI, chest binding, oophorectomy status, and estimated weekly testosterone dose. Confidence interval, CI

In sensitivity analyses, the association between every 6 months of TT and % fibrous stroma achieved significance when alcohol consumption was added as a covariate in the fully adjusted model 3 (exp(β) = 0.99, 95% CI 0.98,1.00, adj *p* = 0.049; Additional file 1: 2) and when cases with atypical lesions were excluded (model 3 exp(β) = 0.99, 95% CI 0.98,1.00, adj *p* = 0.04; Additional file 1: 3). Findings were unaltered when restricting to users that administered TT via intra-muscular injections and non-TT users (Additional file 1: 4). The inverse association between per 6 months of TT use and % epithelium was strengthened when restricted to nulliparous subjects—% epithelium decreased by 4% (model 3 exp(β) = 0.96, 95% CI 0.92,0.99, adj *p* = 0.02); no association between % fibrous stroma or % fat (Additional file 1: 5). When stratified by BMI, the association between per 6 months of TT use and % epithelium remained similar in normal weight subjects (BMI < 25; model 3 exp(β) = 0.96, 95% CI 0.93,1.00, adj* p* = 0.05) but the association was attenuated in overweight/obese subjects (BMI ≥ 25; exp(β) = 0.98, 95% CI 0.95,1.01, adj *p* = 0.14; Additional file 1: 6).

Secondary analyses showed that TT users have 28% less epithelium compared to non-TT users (model 3 exp(β) = 0.72, 95% CI 0.58,0.90, adj *p* = 0.003; Additional file 1: 7). There was no association between TT use and % fibrous stroma or % fat (Additional file 1: 7).

### Subjects with radiology data

The demographics of the 42 subjects who had pre-operative mammography resembled the larger cohort of 425 subjects (Additional file 1: 8). The median age in this subset was 43 years (range 20–61). Six subjects (14.3%) were assessed by the radiologist as having A-fatty breasts, 13 (31.0%) had B-scattered fibroglandular densities, 18 (42.9%) had C-heterogeneously dense breasts, and five (11.9%) had D-extremely dense breasts. The median time between mammogram to surgery was 4.4 months (range 0.4–34.9). Subjects with and without available digital mammogram DICOM files were similar with respect to their demographics (Additional file 1: 8).

### Association of TT and mammographic breast density

There was no correlation between duration of TT at the time of mammography and the radiologist’s breast tissue density assessment (*p* = 0.58; Fig. 3b), and when stratified by age (< 40 years old *n* = 20, rho = − 0.23, 95% CI − 0.58, 0.20, *p* = 0.33; ≥ 40 years old *n* = 22, rho = 0.10, 95% CI − 0.41, 0.51, *p* = 0.65). Among the 25 subjects with DICOM files, LIBRA measurements significantly correlated with the radiologist’s assessment (all *p* ≤ 0.003; Additional file 1: 9), validating the LIBRA measurements. Despite the larger effect sizes, there was no association between every 6 months of TT and any of the LIBRA measurements (*p* > 0.05; Additional file 1: 10), and when stratified by BMI (Additional file 1: 11) or restricted to nulliparous women (Additional file 1: 12). The lack of statistical power in those analyses was due to the small sample size.

### Radiological-pathological correlations

There was no correlation between % epithelium and LIBRA measurements (*p* > 0.05; Additional file 1: 13). However, % fibrous stroma was significantly and positively correlated with both PD (*p* = 0.001) and absolute dense area (*p* < 0.001), but not with absolute non-dense area (*p* = 0.15; Additional file 1: 13). The amount of fat tissue was significantly inversely correlated with PD (*p* = 0.001) and absolute dense area (*p* < 0.001), but not with non-dense area (*p* = 0.14; Additional file 1: 13). The results were similar in sensitivity analysis when restricted to individuals who had surgeries within 6 months after their mammograms (*n* = 17; Additional file 1: 14).

## Discussion

The effect of gender-affirming TT on BC risk is unclear. This study investigated the effect of TT on breast tissue composition in TMIs. In a subset of 42 subjects, we also explored the relationship between TT and mammographic breast density. Leveraging on our deep-learning technology, we demonstrated a quantitative inverse relationship between TT and the amount of breast epithelium; TT did not affect the amount of fibrous stroma and fat. We did not observe any relationship between TT and mammographic breast density. We previously investigated the association of automated breast tissue composition and BC risk in the Nurses’ Health Studies [28]. Women in the highest quartile for % epithelium had higher BC risk compared to women in the lowest quartile; there was no relationship between % fibrous stroma and BC risk [28]. Taken together, our current study provided quantitative histological evidence to support prior epidemiological reports that TT may reduce BC risk compared to cisgender women [3–5].

This study showed that every 6 months of TT use was significantly associated with 3% decrease in the amount of breast epithelium. The small effect size was likely due to the narrow data range (minimum 1.4% to maximum 23.5%; see Additional file 1: 1A). We previously reported that TT exposure of at least 12 months led to alterations in breast morphology [23]. The proportion of trans masculine breast tissues with moderate/marked lobular atrophy increased from 32.9% (non-TT users) to 40.3% (used TT for < 12 month), and to 58.3% (used TT for ≥ 12 months) [23]. Our collective findings suggested that the transition from each lobular atrophy category when assessed by pathologists—minimum, mild, moderate, and marked—may reflect at least 6% decrease in the amount of breast epithelium between each category.

The effect of TT on reducing the amount of breast epithelium was less pronounced in overweight/obese subjects. The effect of TT on breast epithelium may be offset by the endocrine activity of adipose tissue, and that TT may modulate trans masculine BC risk differently in overweight/obese subjects compared to normal weight subjects. The interaction between TT and obesity-related endocrine activity was observed in another of our previous work whereby Toker cell hyperplasia was more frequently detected in overweight/obese subjects using TT than in normal weight subjects [24]. More studies are needed to understand the complex relationship between testosterone, obesity, and BC risk in the trans masculine population.

De Blok et al. estimated the lifetime BC risk for TMIs to be ≈ 3.8%, which was lower than the risk in cisgender women (12%) but remained much higher than the risk in cisgender men (< 0.1%) [14]. BC risk factors for TMIs are not established. While it can be assumed that BC risk factors for TMIs are similar to cisgender women [30], their risk may be modified by TT or chest-contouring surgery. The primary goal of chest-contouring surgery, in contrast to oncologic mastectomies, is the creation of a male-appearing chest rather than the removal of all grossly identifiable breast tissue. In this aspect, chest-contouring surgery resembles reduction mammoplasty. Residual breast tissue after chest-contouring surgery remains hormonally responsive, and BC can still occur [14, 34, 35]. Therefore, even though testosterone is widely reported to have an anti-proliferative effect in the breast [36, 37], TMIs who had chest-contouring surgeries retain their inherent BC risk and TT could modulate that risk. The prevalence of atypical lesions and DCIS in our subjects was lower than that observed in cisgender reduction mammoplasties studies (2% vs. 8%) [20, 38, 39], yet atypical lesions and DCIS were only found in TT users compared with non-users [23]. Our work supports more studies regarding TT in breast pathology, and using preclinical models to understand the extent to which TT affects BC risk in genetically-predisposed individuals [40]

No study has investigated the association between the duration of TT and mammographic breast density in TMIs. Therefore, we attempted to gain preliminary insights into that relationship. We had limited mammography data because of socioeconomic and biobehavioral challenges faced by this population. Excluding young age (< 40 years old), mammography is not frequently performed in TMIs due to many factors such as lack of evidence-based screening guidelines, lack of insurance, poor access to medical care, or quite simply, reluctance. It can be emotionally distressing for TMIs to undergo screening for breast and other “female” cancers because of the discordance between their gender identity and their organ inventory, as well as the feminized language around those procedures and being misgendered in clinics. Nevertheless, the distribution of breast tissue density assessed by the radiologist among the subset of 42 TMIs was similar to the general female population [41]. Our radiological-pathological correlations agreed with previous work that mammographic breast density is mostly contributed by fibrous stroma [42, 43].

Davis et al. randomized 250 postmenopausal women to receive placebo, 150 µg/day, or 300 µg/day testosterone transdermal patch, and observed that PD and absolute dense area were not different between paired baseline and week 52 mammograms [44]. While the null relationship between TT use and mammographic breast density in our study could be explained by insufficient statistical power, it is possible that the decreases in epithelium and stroma were not large enough to be detected via mammograms. More studies with larger sample sizes and intra-subject mammograms are warranted for definitive insights into the relationship between TT and BC risk. Understanding that relationship will have great clinical impact on BC screening strategies for TMIs and reducing their healthcare disparities.

The strengths of our study included leveraging a large study population with comprehensive pathological review and digital slides to understand trans masculine breast tissue composition as it relates to BC risk. We also demonstrated strong correlations between pathologists/radiologist-based assessment and the corresponding computer measures, reiterating the validity and utility of those automated methodologies for large-scale epidemiological research. Our study’s limitations includes pathologists not being blinded to the gender identity of the population sample which could introduce bias, and we were unable to accurately account for TT dosages as dosages are frequently adjusted according to how the subject feels [30]. We only had mammography data in a small subject of 42 subjects and did not have intra-individual mammograms to compare breast density before and after TT use. We also did not have BMI data at the time of mammography. However, BMI was likely to be similar at mammography and surgery as the time between those two events was relatively short (0.4–34.9 months). Lastly, we were unable to control for endogenous estradiol in our analyses. We did not have circulating estradiol levels in our subjects as clinicians do not routinely monitor their estradiol levels. TMIs taking testosterone for at least 6 months tend not to have menstrual cycles, though a small subset can continue to have bleeding and possibly ovulation.

In conclusion, TT decreases breast epithelium, supporting epidemiological findings that TMIs receiving TT, particularly those with normal BMI, may have lower BC risk compared to cisgender women. More studies are needed to investigate the effect of TT on breast density and BC risk in the trans masculine population.

### Supplementary Information

Additional file 1. Supplementary 1 A. Automated breast tissue composition significantly correlated with pathologists’ assessments. The percentage (%) of epithelium (A) significantly inversely correlated with increasing degrees of lobular atrophy (p < 0.001; Spearman’s rho). Cases where the pathologists classified the stroma as predominantly fibrous or fatty were significantly correlated with higher % of fibrous stroma (B; p < 0.001) or fat (C ; p < 0.001), respectively. Each box displays the median, and 25 th and 75 th percentiles (upper and lower hinges). The lower whisker represents the smallest observation greater than or equal to the lower hinge - 1.5 * inter quartile range (IQR); the upper whisker represents the largest observation less than or equal to upper hinge + 1.5 * IQR. B. Automated breast tissue composition remained significantly correlated with pathologists’ assessments even when stratified by testosterone therapy (TT) (all p < 0.001). Spearman’s rho, 95% confidence interval (CI), and p -values comparing the percentage (%) of each tissue region and pathologists’ assessments in A , B , and C , are displayed in D . Each box displays the median, and 25 th and 75 th percentiles (upper and lower hinges). The lower whisker represents the smallest observation greater than or equal to the lower hinge − 1.5 * inter quartile range (IQR); the upper whisker represents the largest observation less than or equal to upper hinge + 1.5 * IQR. Supplementary 2. The association of testosterone therapy (per six months duration) and the percentages (%) of each breast tissue region, additionally adjusting for alcohol consumption in the fully adjusted model 3. Supplementary 3. The association of testosterone therapy (per six months duration) and the percentages (%) of each breast tissue region after excluding the nine subjects with atypical lesions. Supplementary 4. The association of testosterone therapy (per six months duration) and the percentages (%) of each breast tissue region among users who administered testosterone via intra-muscular injection and non-testosterone users. Supplementary 5. The association of testosterone therapy (per six months duration) and the percentages (%) of each breast tissue region and among nulliparous subjects. Supplementary 6. The association of testosterone therapy (per six months duration) and the percentages (%) of each breast tissue region, stratified by body mass index (BMI). Supplementary 7. The association of testosterone therapy use (users/non-users) and the percentages (%) of each breast tissue region. Supplementary 8. Characteristics of 42 transmasculine individuals who had mammography prior to chest contouring surgery. Supplementary 9. Laboratory for Individualized Breast Radiodensity Assessment (LIBRA) breast percent density (PD; B ), absolute dense area (C), and absolute non-dense area (D) significantly correlated with the radiologist’s breast tissue density assessment. Each box displays the median, and 25 th and 75 th percentiles (upper and lower hinges). The lower whisker represents the smallest observation greater than or equal to the lower hinge − 1.5 * inter quartile range (IQR); the upper whisker represents the largest observation less than or equal to upper hinge + 1.5 * IQR. Confidence interval; CI. Supplementary 10. The association between testosterone therapy (per six months duration) and Laboratory for Individualized Breast Radiodensity Assessment (LIBRA) measures. Supplementary 11. The association between testosterone therapy (per six months duration) and Laboratory for Individualized Breast Radiodensity Assessment (LIBRA) measures, stratified by body mass index (BMI). Supplementary 12. The association between testosterone therapy (per six months duration) and Laboratory for Individualized Breast Radiodensity Assessment (LIBRA) measures among nulliparous subjects. Supplementary 13. Scatterplot matrix correlating automated breast tissue composition (pink boxes) with Laboratory for Individualized Breast Radiodensity Assessment (LIBRA) measures (grey boxes) among 23 subjects. Supplementary 14. Scatterplot matrix correlating automated breast tissue composition data (pink boxes) with Laboratory for Individualized Breast Radiodensity Assessment (LIBRA) measures (grey boxes) among 17 subjects who had their chest-contouring surgeries within six months of their mammography.

## Data Availability

The source code for our deep learning networks is available at https://github.com/avellal14/BBD_Pipeline. The data that support the findings of this study are available from Dr. Jan Heng.
