# Supplementary material for: Effect of testosterone therapy on breast tissue composition and mammographic breast density in trans masculine individuals
Source: Breast Cancer Res. 2024 Jul 2;26:109. doi: 10.1186/s13058-024-01867-w (PMC11221014; doi:10.1186/s13058-024-01867-w)

Supplementary 1A

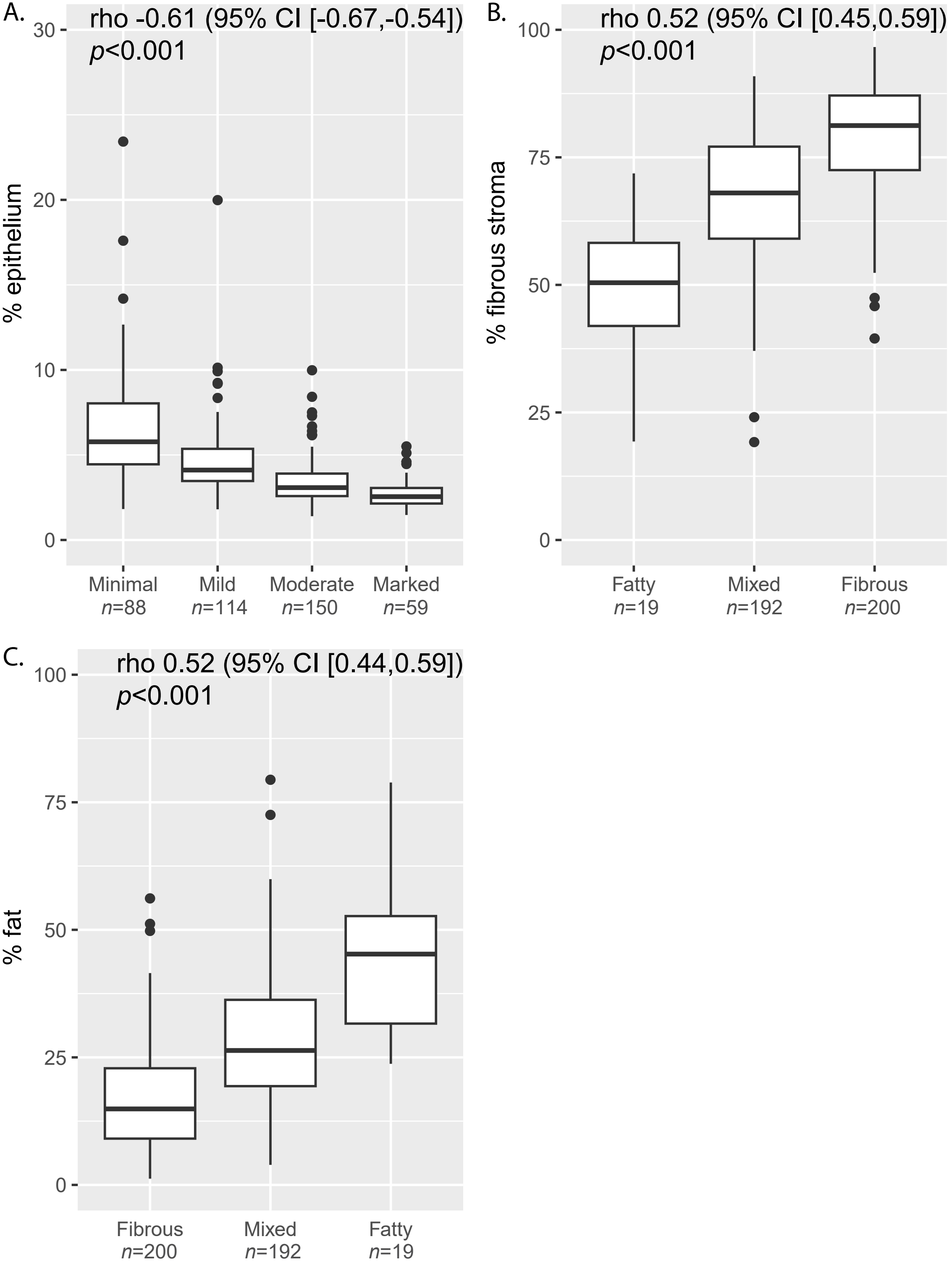

Supplementary 1B

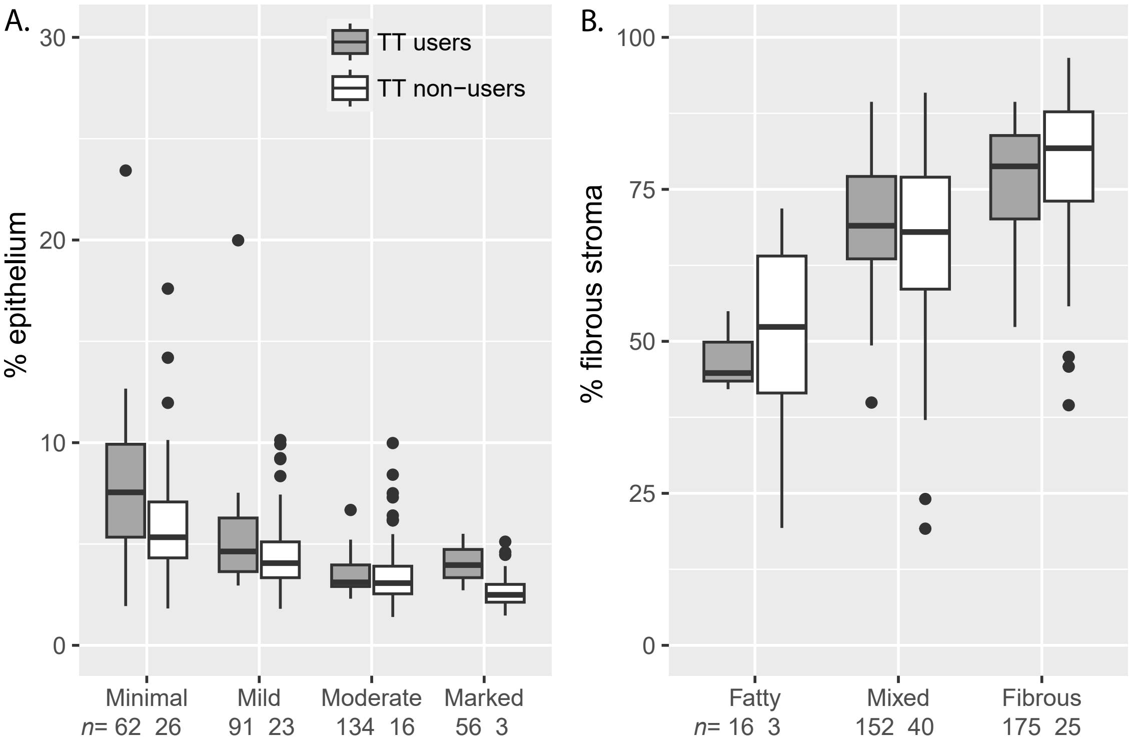

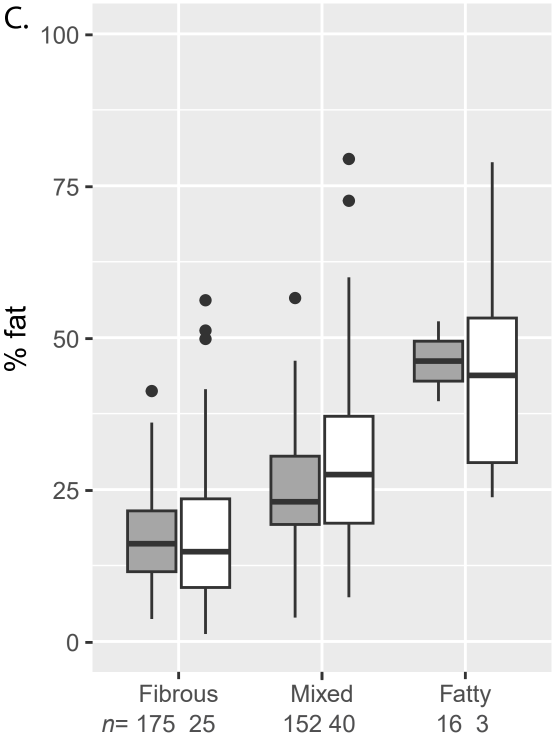

D.

	TT users	
	**rho [95% CI]**	***p* value**
**% epithelium**	-0.59 [-0.66,-0.51]	<0.001
**% fibrous stroma**	0.54 [0.46,0.61]	<0.001
**% fat**	0.53 [0.45,0.60]	<0.001
	**TT non-users**	
	**rho [95% CI]**	***p* value**
**% epithelium**	-0.60 [-0.74,-0.42]	<0.001
**% fibrous stroma**	0.42 [0.18,0.61]	<0.001
**% fat**	0.47 [0.26,0.66]	<0.001

Supplementary 2. The association of testosterone therapy (per six months duration) and the percentages (%) of each breast tissue region, additionally adjusting for alcohol consumption in the fully adjusted model 3.

	n	**Exp(β)**	**95% CI**	***p* value**
**% epithelium**				
Model 1	411	0.97	0.95,0.98	<0.001
Model 2	207	0.96	0.94,0.98	0.001
Model 3	193	0.97	0.95,0.99	0.005
Model 3*	193	0.97	0.94,0.99	0.004
**% fibrous stroma**				
Model 1	411	0.99	0.98,0.99	<0.001
Model 2	207	0.99	0.98,1.00	0.02
Model 3	193	0.99	0.98,1.00	0.05
Model 3*	193	0.99	0.98,1.00	0.049
**% fat**				
Model 1	411	1.02	1.00,1.04	0.01
Model 2	207	1.02	0.99,1.06	0.15
Model 3	193	1.01	0.98,1.05	0.39
Model 3*	193	1.02	0.98,1.05	0.34

Model 1 adjusted for age and year of surgery. Model 2 adjusted for age and year of surgery, race/ethnicity, BMI, chest binding, and oophorectomy status. Model 3 adjusted for age and year of surgery, race/ethnicity, BMI, chest binding, oophorectomy status, and estimated weekly testosterone dose. Confidence interval, CI. *Model 3 additionally adjusting for alcohol consumption.

Supplementary 3. The association of testosterone therapy (per six months duration) and the percentages (%) of each breast tissue region after excluding the nine subjects with atypical lesions.

		n	**Exp(β)**	**95% CI**	***p* value**
% epithelium					
	Model 1	402	0.97	0.95,0.98	<0.001
	Model 2	203	0.96	0.94,0.98	0.001
	Model 3	189	0.97	0.95,0.99	0.007
% fibrous stroma					
	Model 1	402	0.99	0.98,0.99	<0.001
	Model 2	203	0.99	0.98,1.00	0.01
	Model 3	189	0.99	0.98,1.00	0.04
% fat					
	Model 1	402	1.02	1.01,1.04	0.01
	Model 2	203	1.03	0.99,1.06	0.12
	Model 3	189	1.02	0.98,1.05	0.35

Model 1 adjusted for age and year of surgery. Model 2 adjusted for age and year of surgery, race/ethnicity, BMI, chest binding, and oophorectomy status. Model 3 adjusted for age and year of surgery, race/ethnicity, BMI, chest binding, oophorectomy status, and estimated weekly testosterone dose. Confidence interval, CI.

Supplementary 4. The association of testosterone therapy (per six months duration) and the percentages (%) of each breast tissue region among users who administered testosterone via intra-muscular injection and non-testosterone users.

		n	Exp(β)	95% CI	*p* value
% epithelium					
	Model 1	369	0.96	0.95,0.97	<0.001
	Model 2	185	0.96	0.94,0.98	<0.001
	Model 3	174	0.97	0.94,0.99	0.01
% fibrous stroma					
	Model 1	369	0.98	0.98,0.99	<0.001
	Model 2	185	0.99	0.98,1.00	0.04
	Model 3	174	0.99	0.98,1.00	0.07
% fat					
	Model 1	369	1.03	1.00,1.05	0.02
	Model 2	185	1.02	0.98,1.05	0.34
	Model 3	174	1.02	0.98,1.05	0.42

Model 1 adjusted for age and year of surgery. Model 2 adjusted for age and year of surgery, race/ethnicity, BMI, chest binding, and oophorectomy status. Model 3 adjusted for age and year of surgery, race/ethnicity, BMI, chest binding, oophorectomy status, and estimated weekly testosterone dose. Confidence interval, CI.

Supplementary 5. The association of testosterone therapy (per six months duration) and the percentages (%) of each breast tissue region among nulliparous subjects.

		n	**Exp(β)**	**95% CI**	***p* value**
% epithelium					
	Model 1	171	0.97	0.95,0.98	0.001
	Model 2	87	0.96	0.93,0.99	0.02
	Model 3	82	0.96	0.92,0.99	0.02
% fibrous stroma					
	Model 1	171	1.00	0.99,1.01	0.75
	Model 2	87	0.99	0.98,1.01	0.27
	Model 3	82	0.99	0.98,1.01	0.29
% fat					
	Model 1	171	1.01	0.98,1.04	0.49
	Model 2	87	1.02	0.98,1.07	0.32
	Model 3	82	1.01	0.97,1.06	0.49

Model 1 adjusted for age and year of surgery. Model 2 adjusted for age and year of surgery, race/ethnicity, BMI, chest binding, and oophorectomy status. Model 3 adjusted for age and year of surgery, race/ethnicity, BMI, chest binding, oophorectomy status, and estimated weekly testosterone dose. Confidence interval, CI.

Supplementary 6. The association of testosterone therapy (per six months duration) and the percentages (%) of each breast tissue region, stratified by body mass index (BMI).

		n	Exp(β)	95% CI	*p* value
**BMI <25**					
% epithelium					
	Model 1	177	0.94	0.92,0.97	<0.001
	Model 2	98	0.95	0.92,0.98	0.004
	Model 3	91	0.96	0.93,1.00	0.05
% fibrous stroma					
	Model 1	177	1.00	0.99,1.01	0.64
	Model 2	98	1.00	0.99,1.01	0.60
	Model 3	91	1.00	0.99,1.01	0.95
% fat					
	Model 1	177	1.01	0.97,1.05	0.54
	Model 2	98	1.03	0.97,1.09	0.32
	Model 3	91	1.02	0.96,1.09	0.53
**BMI ≥25**					
% epithelium					
	Model 1	232	0.97	0.96,0.99	<0.001
	Model 2	109	0.98	0.95,1.00	0.10
	Model 3	102	0.98	0.95,1.01	0.14
% fibrous stroma					
	Model 1	232	0.98	0.98,0.99	<0.001
	Model 2	109	0.98	0.97,1.00	0.04
	Model 3	102	0.99	0.97,1.00	0.08
% fat					
	Model 1	232	1.02	1.00,1.03	0.06
	Model 2	109	1.01	0.98,1.05	0.46
	Model 3	102	1.00	0.96,1.04	0.97

Model 1 adjusted for age and year of surgery. Model 2 adjusted for age and year of surgery, race/ethnicity, chest binding, and oophorectomy status. Model 3 adjusted for age and year of surgery, race/ethnicity, chest binding, oophorectomy status, and estimated weekly testosterone dose. Confidence interval, CI.

Supplementary 7. The association of testosterone therapy use (users/non-users) and the percentages (%) of each breast tissue region.

		n	**Exp(β)**	**95% CI**	***p* value**
% epithelium					
	Model 1	425	0.70	0.63,0.79	<0.001
	Model 2	209	0.68	0.57,0.83	<0.001
	Model 3	195	0.72	0.58,0.90	0.003
% fibrous stroma					
	Model 1	425	1.00	0.94,1.06	0.96
	Model 2	209	0.97	0.89,1.05	0.41
	Model 3	195	1.01	0.92,1.10	0.89
% fat					
	Model 1	425	1.01	0.84,1.21	0.94
	Model 2	209	1.04	0.79,1.36	0.80
	Model 3	195	0.90	0.66,1.22	0.49

Model 1 adjusted for age and year of surgery. Model 2 adjusted for age and year of surgery, race/ethnicity, BMI, chest binding, and oophorectomy status. Model 3 adjusted for age and year of surgery, race/ethnicity, BMI, chest binding, oophorectomy status, and estimated weekly testosterone dose. Confidence interval, CI.

Supplementary 8. Characteristics of 42 transmasculine individuals who had mammography prior to chest contouring surgery.

	**All Individuals**	**Individuals with DICOM files**	**Individuals without DICOM files**	***p* value**
**N (%)**	42	25	17	
**Age at mammogram, median [IQR]**	43.3 [37.8, 48.5]	38.8 [30.6, 45.5]	46.1 [39.5, 49.3]	0.09[a]
**Race/ethnicity, *n* (%)**				0.35[b]
White	33 (78.6)	18 (72.0)	15 (88.2)	
Black or African American	6 (14.3)	5 (20.0)	1 (5.9)	
Asian	1 (2.4)	0 (0.0)	1 (5.9)	
Multiracial	1 (2.4)	1 (4.0)	0 (0.0)	
Native American/Pacific Islander	1 (2.4)	1 (4.0)	0 (0.0)	
**Family history of breast cancer, *n* (%)**				0.33[b]
Yes	13 (31.0)	9 (36.0)	4 (23.5)	
No	27 (64.3)	14 (56.0)	13 (76.5)	
Not reported	2 (4.8)	2 (8.0)	0 (0.0)	
**Oophorectomy prior to mammogram, *n* (%)**				1.00[b]
Yes	5 (11.9)	3 (12.0)	2 (11.8)	
No	37 (88.1)	22 (88.0)	15 (88.2)	
**BMI at surgery, median [IQR]**	28.5 [24.6, 30.0]	28.7 [25.7, 30.1]	26.5 [24.1, 29.8]	0.51[a]
**Duration of testosterone therapy at mammogram, *n* (%)**				0.45[b]
Never	9 (21.4)	3 (12.0)	6 (35.3)	
<1 year	8 (19.0)	5 (20.0)	3 (17.6)	
>=1 to <2 years	15 (35.7)	10 (40.0)	5 (29.4)	
>=2 to <5 years	9 (21.4)	6 (24.0)	3 (17.6)	
>=5 years	1 (2.4)	1 (4.0)	0 (0.0)	
**Chest binding at mammogram, *n* (%)**				1.00[b]
Yes	19 (45.2)	16 (94.1)	3 (100.0)	
No	1 (2.4)	1 (5.9)	0 (0.0)	
Not reported	22 (52.4)	3 (12.0)	3 (17.6)	
**Breast tissue density assessed by the radiologist**				0.48[b]
A-Fatty	6 (14.3)	3 (12.0)	3 (17.6)	
B-Scattered fibroglandular	13 (31.0)	7 (28.0)	6 (35.3)	
C-Heterogeneously dense	18 (42.9)	13 (52.0)	5 (29.4)	
D-Dense	5 (11.9)	2 (8.0)	3 (17.6)	

p-values were obtained using the [a]Mann-Whitney or [b]Fisher's exact test by comparing those that had DICOM files versus those that did not. Data in "not reported" categories were excluded from statistical analysis. Body mass index, BMI; Inter-quartile range, IQR. Percentages may not add up to 100% due to rounding.

Supplementary 9

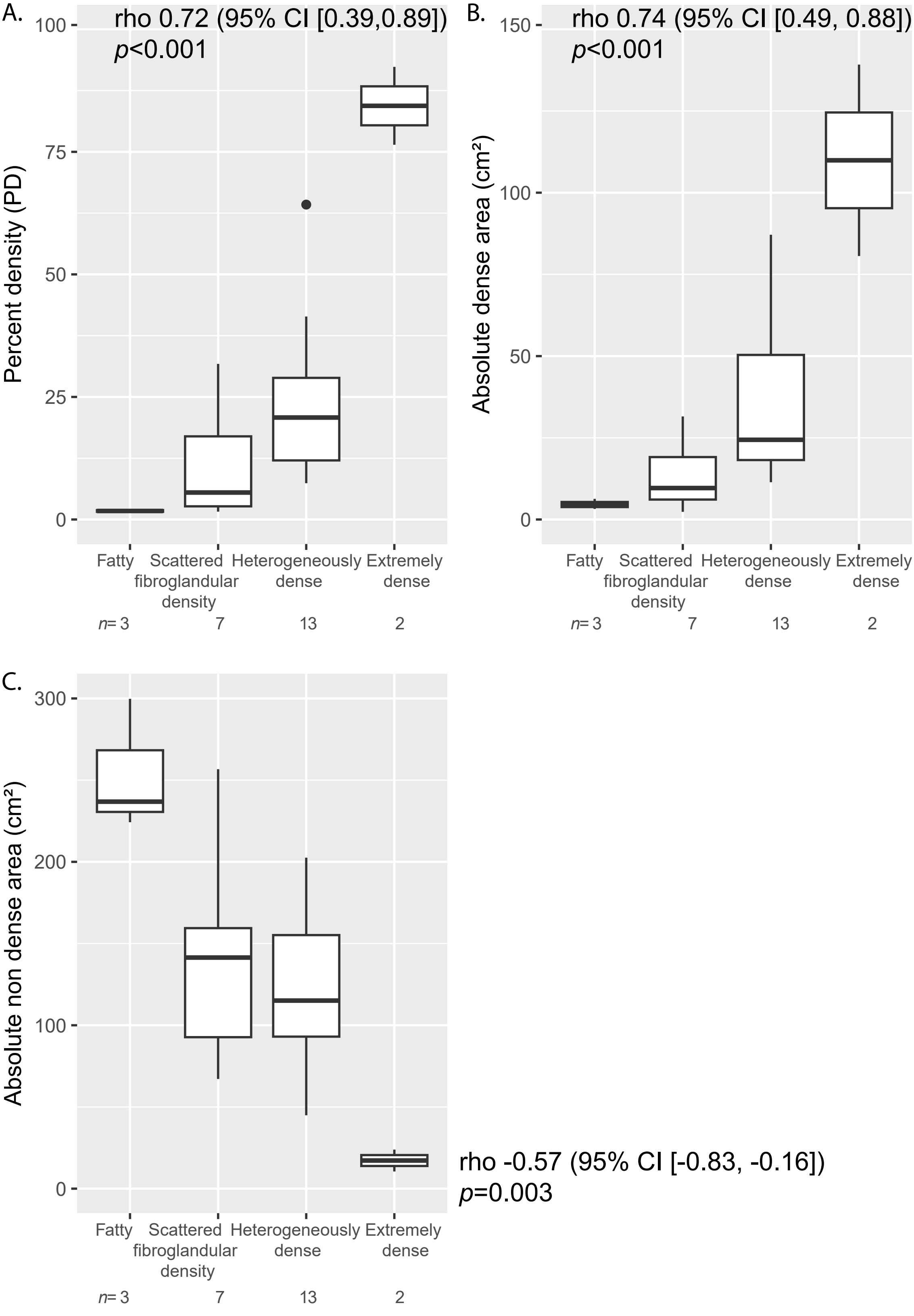

Supplementary 10. The association between testosterone therapy (per six months duration) and LIBRA measures.

	n	Exp(β)	95% CI	*p* value
**Percent density**				
Crude	25	0.89	0.76,1.06	0.18
Adjusted model	25	0.97	0.83,1.14	0.71
**Absolute dense area**				
Crude	25	0.90	0.77,1.04	0.14
Adjusted model	25	0.94	0.81,1.09	0.41
**Absolute non-dense area**				
Crude	25	1.05	0.95,1.17	0.31
Adjusted model	25	1.00	0.92,1.08	0.92

The adjusted model included age at mammogram and BMI at chest-contouring surgery.

Supplementary 11. The association between testosterone therapy (per six months duration) and LIBRA measures, stratified by body mass index (BMI).

	n	**Exp(β)**	**95% CI**	***p* value**
**BMI <25**				
**Percent density**				
Crude	6	1.06	0.68,1.65	0.74
Adjusted model	6	1.09	0.67,1.78	0.61
**Absolute dense area**				
Crude	6	0.96	0.63,1.47	0.80
Adjusted model	6	0.98	0.60,1.60	0.93
**Absolute non-dense area**				
Crude	6	0.92	0.67,1.27	0.52
Adjusted model	6	0.90	0.64,1.26	0.40
**BMI≥25**				
**Percent density**				
Crude	19	0.89	0.74,1.08	0.23
Adjusted model	19	0.88	0.72,1.08	0.21
**Absolute dense area**				
Crude	19	0.90	0.75,1.08	0.23
Adjusted model	19	0.88	0.73,1.07	0.19
**Absolute non-dense area**				
Crude	19	1.06	0.94,1.18	0.32
Adjusted model	19	1.08	0.96,1.21	0.18

The adjusted model included age at mammogram only.

Supplementary 12. The association between testosterone therapy (per six months duration) and LIBRA measures among nulliparous subjects.

	n	Exp(β)	95% CI	*p* value
**Percent density**				
Crude	11	0.82	0.64,1.05	0.10
Adjusted model	11	0.88	0.67,1.16	0.31
**Absolute dense area**				
Crude	11	0.82	0.66,1.02	0.07
Adjusted model	11	0.86	0.67,1.11	0.21
**Absolute non-dense area**				
Crude	11	1.08	0.90,1.29	0.37
Adjusted model	11	1.06	0.92,1.22	0.35

The adjusted model included age at mammogram and BMI at chest-contouring surgery.

Supplementary 13

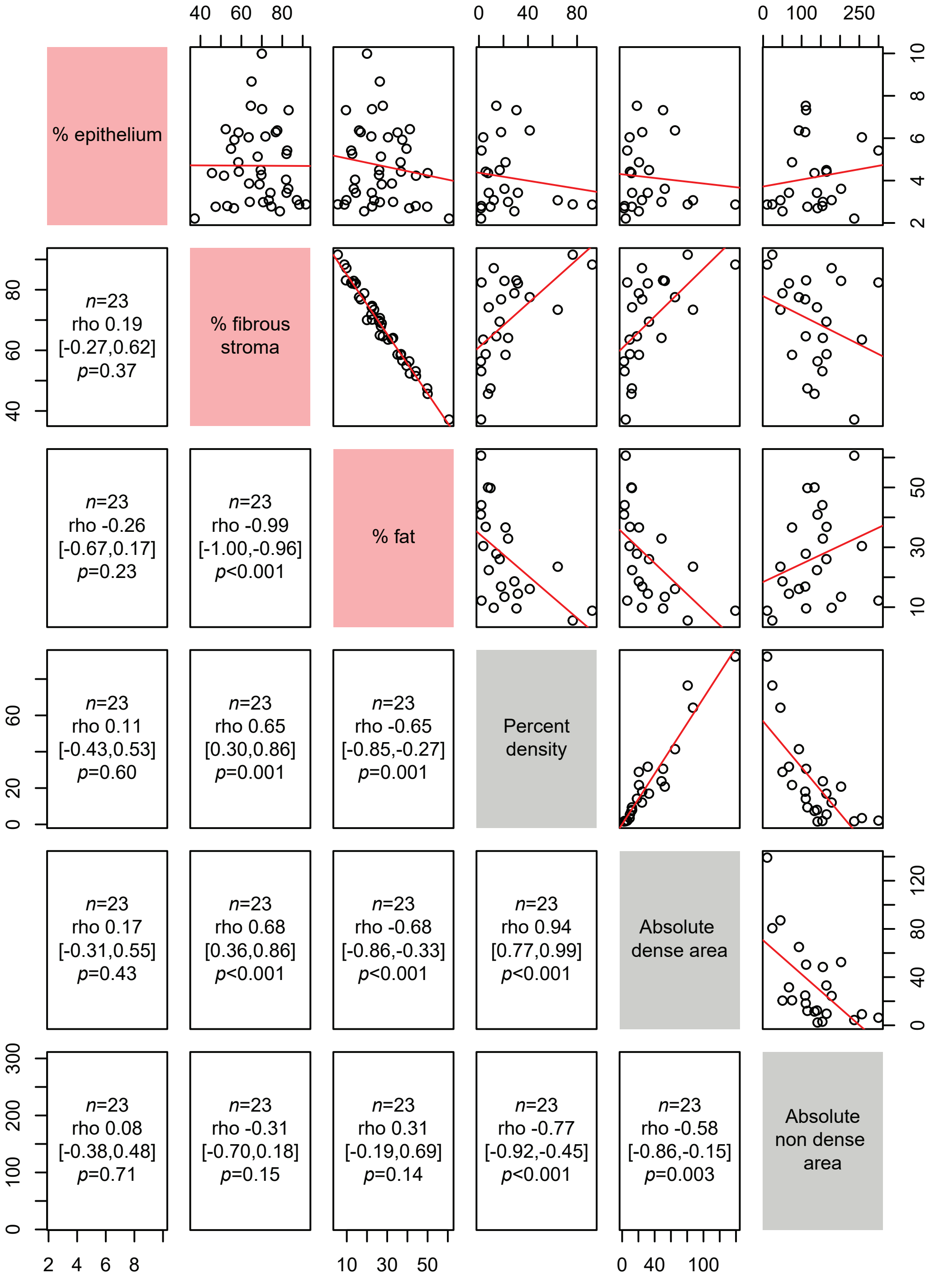

Supplementary 14

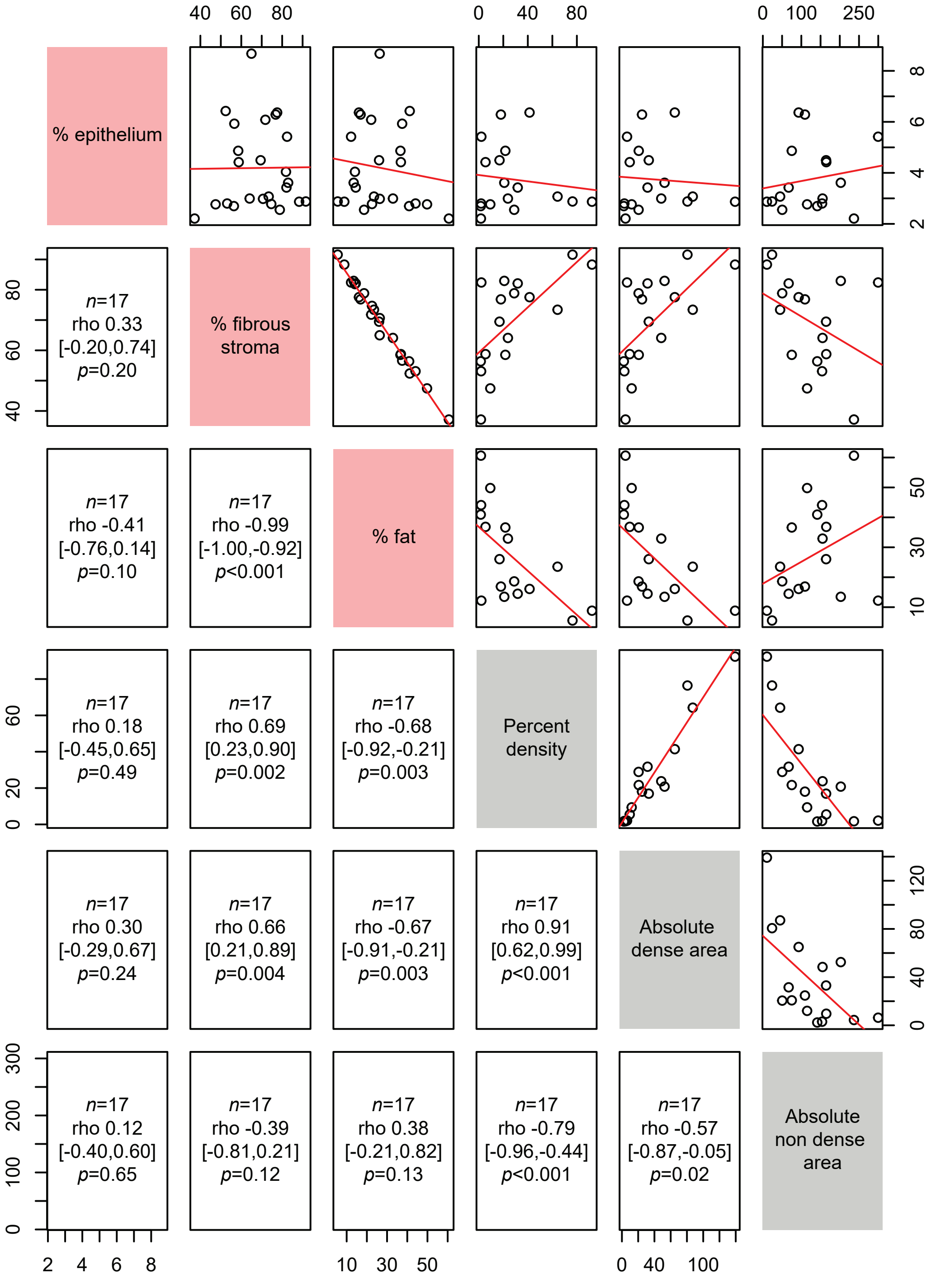

Supplement: Supplementary file 1 — Additional file 1. Supplementary 1 A. Automated breast tissue composition significantly correlated with pathologists’ assessments. The percentage (%) of epithelium (A) significantly inversely correlated with increasing degrees of lobular atrophy (p < 0.001; Spearman’s rho). Cases where the pathologists classified the stroma as predominantly fibrous or fatty were significantly correlated with higher % of fibrous stroma (B; p < 0.001) or fat (C ; p < 0.001), respectively. Each box displays the median, and 25 th and 75 th percentiles (upper and lower hinges). The lower whisker represents the smallest observation greater than or equal to the lower hinge - 1.5 * inter quartile range (IQR); the upper whisker represents the largest observation less than or equal to upper hinge + 1.5 * IQR. B. Automated breast tissue composition remained significantly correlated with pathologists’ assessments even when stratified by testosterone therapy (TT) (all p < 0.001). Spearman’s rho, 95% confidence interval (CI), and p -values comparing the percentage (%) of each tissue region and pathologists’ assessments in A , B , and C , are displayed in D . Each box displays the median, and 25 th and 75 th percentiles (upper and lower hinges). The lower whisker represents the smallest observation greater than or equal to the lower hinge − 1.5 * inter quartile range (IQR); the upper whisker represents the largest observation less than or equal to upper hinge + 1.5 * IQR. Supplementary 2. The association of testosterone therapy (per six months duration) and the percentages (%) of each breast tissue region, additionally adjusting for alcohol consumption in the fully adjusted model 3. Supplementary 3. The association of testosterone therapy (per six months duration) and the percentages (%) of each breast tissue region after excluding the nine subjects with atypical lesions. Supplementary 4. The association of testosterone therapy (per six months duration) and the percentages (%) of each breast tissu [file 13058_2024_1867_MOESM1_ESM.pdf]
